# Linitis Plastica-Like Gastric Carcinoma in a 22-Year-Old South American Migrant: Histologic–Gross Discordance in an Aggressive Diffuse Tumour

**DOI:** 10.7759/cureus.111222

**Published:** 2026-06-21

**Authors:** Hubert Daisley, Nathan Williams, Arlene Rampersad

**Affiliations:** 1 Pathology, San Fernando General Hospital, San Fernando, TTO

**Keywords:** diffuse gastric carcinoma, early-onset gastric cancer, helicobacter pylori, histopathology, linitis plastica, venezuela, young adult

## Abstract

Gastric cancer in young patients is uncommon and frequently presents at an advanced stage with aggressive clinicopathological features. Diffuse gastric carcinoma, including linitis plastica, is characterised by widespread infiltration of the gastric wall and poor prognosis.

We report a 22-year-old Venezuelan woman who presented with vomiting and weight loss and subsequently developed shortness of breath, fatigue, melaena, ascites, and generalised lymphadenopathy. Testing for Helicobacter pylori was performed, which was positive. Despite supportive management, she deteriorated rapidly and died shortly after readmission.

Autopsy revealed diffuse gastric wall thickening with nodular ulcerative mucosa and peritoneal dissemination. Histology demonstrated a diffuse infiltrative carcinoma with prominent desmoplasia and lymph vascular invasion, consistent with a linitis plastica-like growth pattern.

Metastatic carcinoma was present in the lymph nodes and both lungs. In the lungs, the tumour exhibited lepidic spread along the alveolar septae, resulting in consolidation and a pattern mimicking bronchioloalveolar carcinoma.

This case highlights the aggressive behaviour of diffuse gastric carcinoma in young patients and underscores the importance of clinicopathological correlation.

## Introduction

Gastric carcinoma remains a major cause of global cancer-related morbidity and mortality and shows marked geographic variation in incidence [1]. Although it predominantly affects older adults, a clinically important minority of cases occur in younger patients, and these early-onset cases more often present with advanced disease and adverse tumour biology [2,3].

Gastric adenocarcinoma can be broadly divided into intestinal and diffuse types according to Lauren’s classification [4]. Intestinal-type tumours tend to form discrete mass lesions, whereas diffuse-type tumours are characterised by poorly cohesive cells and infiltrative growth that may result in diffuse thickening of the gastric wall [5,6].

Linitis plastica represents an aggressive diffuse gastric carcinoma phenotype associated with circumferential infiltration, desmoplasia, wall thickening, and rigidity [7,8]. Although classically associated with diffuse mural involvement without a discrete mass, variability in gross appearance is recognised [7,9].

Hereditary diffuse gastric cancer is an important diagnostic consideration in young patients with diffuse gastric carcinoma and is most commonly associated with pathogenic CDH1 variants [10,11]. Updated clinical guidelines highlight its relevance even in cases without a clear family history and discuss the clinical role of CDH1 alterations [11]. In addition, E-cadherin loss has recognised relevance in breast pathology, and metastatic lobular breast carcinoma may mimic primary gastric linitis plastica, underscoring the importance of clinicopathological correlation [12-14].

Environmental and infectious factors remain central to gastric carcinogenesis. Helicobacter pylori is a well-established gastric carcinogen [15-17]. The burden of gastric cancer is substantial in Central and South America [18], and Venezuelan studies have demonstrated associations among H. pylori, premalignant lesions, and gastric carcinoma [18]. Additional environmental exposures, including bracken fern, have also been implicated epidemiologically [19].

We report a rapidly fatal case of diffuse gastric carcinoma in a 22-year-old Venezuelan migrant with confirmed H. pylori infection and linitis plastica-like histology.

## Case presentation

A 22-year-old Venezuelan migrant was admitted with a two-month history of persistent vomiting and weight loss. She reported a prior history of pancreatitis treated in her native country. During her initial admission, she received intravenous fluids and proton pump inhibitor therapy but discharged herself against medical advice.

Nine days later, she was readmitted with shortness of breath, fatigue, and passage of melaena stools.

On examination, the patient was cachectic and pale, with no cyanosis or jaundice. Breast and pelvic examinations were unremarkable. Respiratory findings were consistent with right-sided pulmonary consolidation. Ascites was present without palpable abdominal masses. Generalised lymphadenopathy was noted, involving bilateral cervical and inguinal nodes.

Laboratory investigations showed anaemia (haemoglobin 9.7 g/dL; reference range: 11.7-15.5 g/dL) and a white blood cell count of 7.1 × 10³/mL (reference range: 4.10-11.20 × 10³/mL). Testing for Helicobacter pylori was performed, which was positive. Despite supportive management, the patient deteriorated rapidly and died on the second day following readmission.

Autopsy findings

Gross Findings

**Figure 1:**
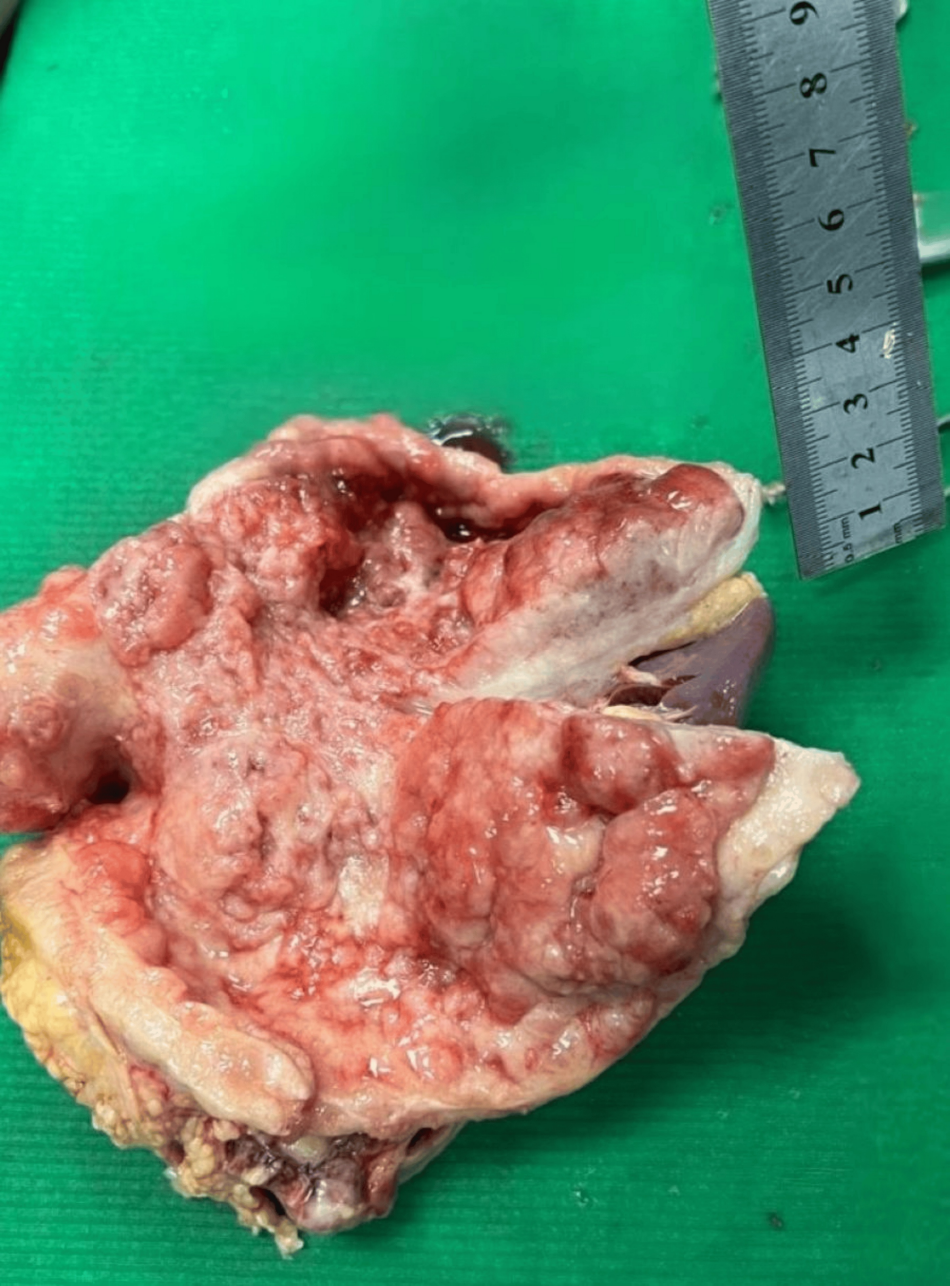
Gross appearance of the stomach with tumour. Gross stomach specimen demonstrating diffuse wall thickening with nodular ulcerative mucosa, loss of rugal folds, and serosal/omental tumour deposits consistent with peritoneal dissemination.

The stomach showed irregular mucosal nodularity with multiple nodules measuring approximately 0.5-4.0 cm and associated ulceration. The gastric wall was markedly and diffusely thickened with loss of normal rugal folds, producing a rigid, indurated appearance. Tumour deposits were present on the serosal surface of the stomach and within the greater omentum, consistent with peritoneal dissemination (Figure 1).

Gross examination of both lungs showed extensive bilateral consolidation. No gross evidence of breast malignancy was identified.

Histopathological Findings

**Figure 2:**
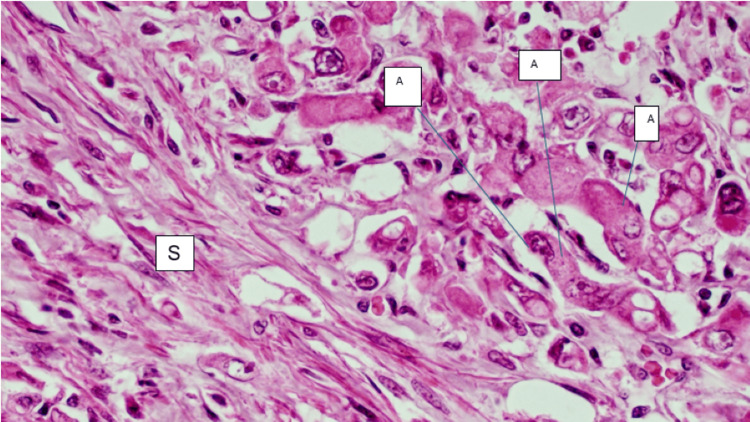
Histology of the stomach neoplasm. Haematoxylin and eosin stain (10×40) demonstrating infiltrative poorly cohesive malignant cells within desmoplastic stroma (S) with lymph vascular invasion (A).

Although gross examination showed nodular ulceration of the gastric mucosa, histological evaluation demonstrated diffuse infiltrative adenocarcinoma composed of poorly cohesive malignant cells infiltrating the gastric wall within a prominent desmoplastic stroma. Extensive lymph vascular invasion was present (Figure 2). This diffuse infiltrative growth pattern was consistent with linitis plastica-like diffuse gastric carcinoma.

Metastatic carcinoma was identified in the cervical lymph nodes and bilaterally in the lungs. Pulmonary involvement consisted of tumour cells spreading along alveolar septae in a lepidic pattern, associated with parenchymal consolidation.

## Discussion

Early-onset gastric carcinoma represents a small but clinically important subset of gastric cancers and is more frequently associated with diffuse histology, advanced disease, and poor outcomes [2,3]. Diffuse gastric carcinoma is characterised by infiltrative growth, early dissemination, and a tendency towards peritoneal and lymphatic spread [2,3,5,6].

In the present case, the gross and microscopic findings support diffuse gastric carcinoma with a linitis plastica-like growth pattern [7-9]. Diffuse wall thickening and rigidity are characteristic of this phenotype, whereas the nodular ulcerative mucosal surface represents recognised morphological variability rather than strict gross-histological discordance [7-9].

If encountered endoscopically, such nodular and ulcerative mucosal changes could broaden the differential diagnosis. However, a thickened and non-compliant gastric wall would remain highly suggestive of an infiltrative diffuse process [7,8].

The confirmed H. pylori positivity in this patient is significant. H. pylori plays a central role in gastric carcinogenesis through chronic inflammation and mucosal injury [15-17]. Regional epidemiological data from Venezuela further support its relevance [18]. Contemporary classification and carcinogenicity frameworks also emphasise inflammation-driven and environmentally mediated pathways in gastric adenocarcinoma development [6,18].

Given the patient’s young age and diffuse tumour phenotype, hereditary diffuse gastric cancer remains an important consideration [10,11]. However, no family history, genetic analysis, or E-cadherin immunohistochemistry was available, and therefore, this diagnosis cannot be confirmed. The broader diagnostic principle remains important because metastatic lobular breast carcinoma may mimic primary diffuse gastric carcinoma [12-14]. No breast tumour was identified in this patient clinically or at autopsy.

The pulmonary findings are particularly noteworthy. Diffuse metastatic spread along alveolar septae produced bilateral consolidation and likely contributed to respiratory compromise. This pattern reflects advanced disease consistent with modern staging frameworks for gastric carcinoma [20].

Overall, this case underscores the importance of considering diffuse gastric carcinoma in young patients with alarming symptoms and highlights the role of histopathology in establishing the diagnosis when macroscopic features are atypical [5,6].

## Conclusions

This case demonstrates a rapidly progressive diffuse gastric carcinoma with linitis plastica-like features in a 22-year-old Venezuelan migrant with confirmed H. pylori infection.

It highlights the aggressive behaviour of diffuse gastric carcinoma in very young patients, the variability that may occur in gross presentation, and the importance of careful histopathological evaluation in establishing an accurate diagnosis. The case also raises the possibility of hereditary diffuse gastric cancer as an underlying predisposition, although this could not be further assessed because family history and molecular studies were unavailable.
